# Generation of 3D Human iPSC-Derived Multi-Cell Type Neurospheres for Studying Neuron, Astrocyte, and Microglia Crosstalk

**DOI:** 10.21769/BioProtoc.5493

**Published:** 2025-11-05

**Authors:** Stefan Wendt, Christopher Lee, Wenji Cai, Ada J. Lin, Jessica Huang, V. Poon, Xianyuan Xiang, Wei Hong, Brian A. MacVicar, Haakon B. Nygaard

**Affiliations:** 1 Djavad Mowafaghian Centre for Brain Health, University of British Columbia, VCR, Canada; 2 Department of Psychiatry, University of British Columbia, VCR, Canada; 3 Division of Neurology, Faculty of Medicine, University of British Columbia, VCR, Canada; 4 Faculty of Life and Health Sciences, Shenzhen University of Advanced Technology, Shenzhen, China; 5 Shenzhen Key Laboratory of Neuroimmunomodulation for Neurological Diseases, Shenzhen-Hong Kong Institute of Brain Science, Shenzhen Institutes of Advanced Technology, Chinese Academy of Sciences, Shenzhen, China

**Keywords:** iPSC-derived cells, 3D neural tissue, Microglia, Neuro–glia interaction, In vitro disease modeling

## Abstract

Three-dimensional (3D) human brain tissue models derived from induced pluripotent stem cells (iPSCs) have transformed the study of neural development and disease in vitro. While cerebral organoids offer high structural complexity, their large size often leads to necrotic core formation, limiting reproducibility and challenging the integration of microglia. Here, we present a detailed, reproducible protocol for generating multi-cell type 3D neurospheres that incorporate neurons, astrocytes, and optionally microglia, all derived from the same iPSCs. While neurons and astrocytes differentiate spontaneously from neural precursor cells, generated by dual SMAD-inhibition (blocking BMP and TGF-b signaling), microglia are generated in parallel and can infiltrate the mature neurosphere tissue after plating neurospheres into 48-well plates. The system supports a range of downstream applications, including functional confocal live imaging of GCaMP6f after adeno-associated virus (AAV) transduction of neurospheres or immunofluorescence staining after fixation. Our approach has been successfully implemented across multiple laboratories, demonstrating its robustness and translational potential for studying neuron–glia interactions and modeling neurodegenerative processes.

Key features

• Reproducible human iPSC-derived 3D neurosphere multi-cell type tissue culture system.

• Optional addition of microglia allows for studying neuron–microglia interaction in vitro in 3D.

• Reliable spontaneous activity offers functional tissue culture readouts of neural firing.

• System allows modeling of human brain diseases, such as Alzheimer’s disease.

## Graphical overview


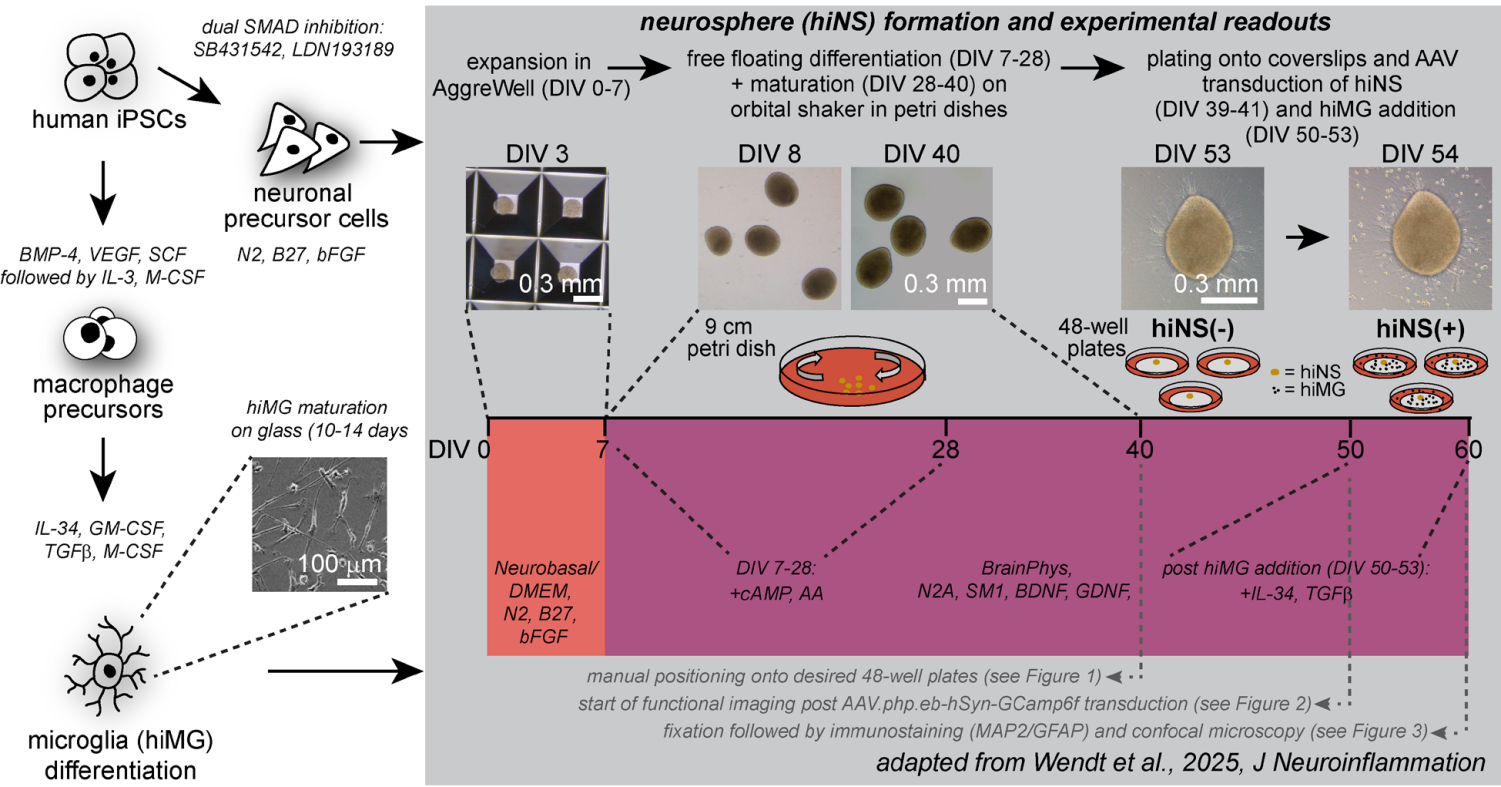


**Graphical overview of human induced pluripotent stem cell (iPSC)-derived 3D neurosphere (hiNS) protocol, first presented in Wendt et al. [14].** Key steps along the protocol, media components, and experimental example readouts are highlighted. The protocol offers a reliable and robust differentiation of human 3D neural tissue comprised of neurons (both excitatory and inhibitory in nature) and astrocytes, as well as optional infiltration of microglial-like cells. This approach is particularly useful for studying microglial impact on neuron and astrocyte function in a controlled 3D tissue culture. Within the Nygaard laboratory at the University of British Columbia, this protocol was successfully used using an in house–generated iPSC line from a healthy individual as well as the commercially available KOLF2.1J [1] (The Jackson Laboratory). In addition, the Kettenmann laboratory at the Shenzhen Institutes of Advanced Technology successfully reproduced the protocol by using an additional healthy control iPSC cell line from the Chinese Academy of Sciences stem cell bank (cell line ID: DYR0100 [2])

## Background

Induced pluripotent stem cells (iPSCs) were first generated by reprogramming somatic cells with defined transcription factors in mice [3], soon extended to human cells [4,5], revolutionizing personalized disease modeling. Early work focused on 2D monolayer cultures, but their limitations in mimicking tissue complexity spurred the development of 3D systems. Breakthroughs such as cerebral organoids [6] (COs) and guided cortical spheroids [7] marked the transition to iPSC-derived 3D cultures that recapitulate human brain architecture and function. Since the first report of 3D human iPSC-derived cerebral organoids for studying human *mini brains* in a dish [6], the organoid research field has grown and advanced substantially. COs are directly derived from iPSCs by forming ectodermal embryoid bodies followed by differentiation protocols into neural lineages. While COs can achieve remarkable tissue complexities, mimicking the embryonic human brain development [8,9], they are well known to form necrotic cores due to their large size, leading to limited oxygen and media diffusion deep within the 3D tissue [10,11]. The presence of necrotic cores is a major challenge for scientists aiming to introduce microglia, the brain resident immune cells, to CO to study their interaction with neural tissue in physiological conditions. An alternative 3D brain tissue culture approach results in the formation of smaller *mini brains*, largely avoiding the formation of necrotic cores: neurospheres. While neurospheres are sometimes referred to as organoids in the literature, their differentiation is distinctly different from most CO differentiation protocols. Most importantly, embryonic stem cells or iPSCs are not differentiated into embryonic bodies but rather differentiated first into neural precursor cells (NPCs) following a dual SMAD inhibition differentiation paradigm [12,13]. In suspension culture, these NPCs form aggregates that can be further differentiated, resulting into neurospheres harboring neuronal cell populations as well as astrocytes. Unlike COs, neurospheres are rapidly producible and amenable to high-throughput experimental designs, making them ideal for use in bridging the gap between mechanistic studies and translational research. We recently developed a reproducible human iPSC-derived 3D neurosphere cell culture protocol, including the addition of iPSC-derived microglia, forming a multi-cell type neural tissue, and used it to further develop a chronic amyloidosis protocol for modeling Alzheimer’s disease [14]. Here, we will provide detailed methods and step-by-step instructions for the generation of our neuron–astrocyte–microglia tri-culture neurospheres as a robust basis for studying neuron–glia interaction in physiology, or for model development for brain pathologies. Our protocol was recently reproduced at the Shenzhen Institutes of Advanced Technology; here, we show example data generated from two laboratories highlighting the reproducibility of this protocol.

## Materials and reagents

**Biological materials**

1. HN1 human iPSC cell line (produced in-house)

2. KOLF2.1J human iPSC cell line (The Jackson Laboratory, catalog number: JIPSC001000)

3. DYR0100 human iPSC cell line (Chinese Academy of Sciences stem cell bank, number SCSP-1301)

**Reagents**

*Note: Details are given for storage conditions. Specifics were not given over aliquot volume for supplements as this will vary depending on the number of experiments/differentiations being performed, and each research group will need to adjust volume to minimize freeze/thaw cycles.*

1. mTeSR^TM^ Plus (mTeSR Plus) (STEMCELL Technologies, catalog number: 100-0276); mTeSR Plus is provided as a base solution (400 mL; store at 4 °C) and supplement (100 mL; store at -20 °C)

2. Y-27632 (dihydrochloride) (ROCK inhibitor) (STEMCELL Technologies, catalog number: 72304); reconstitute in 1.561 mL of sterile dH_2_O to achieve a 10 mM solution and store as aliquots at -20 °C

3. Corning^®^ Matrigel^®^ growth factor reduced (GFR) basement membrane matrix, LDEV-free, 10 mL (Matrigel) (Corning, catalog number: 354230); store at -20 °C

4. PBS, pH 7.4 (ThermoFisher Scientific, catalog number: 10010023); store at 4 °C

5. Dimethyl sulfoxide, Hybri-Max^TM^, sterile, suitable for hybridoma, ≥99.7% (DMSO) (Millipore Sigma, catalog number: D2650-100ML); store at RT

6. Accutase cell detachment solution (Accutase) (Millipore Sigma, catalog number: SCR005); store at 4 °C

7. ReLeSR^TM^ (STEMCELL Technologies, catalog number: 100-0483); store at RT

8. DMEM/F-12 (ThermoFisher Scientific, catalog number: 11320033); store at 4 °C

9. Neurobasal^TM^ medium (neurobasal) (ThermoFisher Scientific, catalog number: 21103049); store at 4 °C

10. B-27^TM^ supplement (50×), serum free (B-27) (ThermoFisher Scientific, catalog number: 17504044); store at -20 °C

11. Cell Therapy Systems N-2 Supplement (N-2) (ThermoFisher Scientific, catalog number: 17502048); store at -20 °C

12. GlutaMAX^TM^ supplement (GlutaMAX) (ThermoFisher Scientific, catalog number: 35050061); store at RT

13. MEM non-essential amino acids solution (100×) (MEM-NEAA) (ThermoFisher Scientific, catalog number: 11140050); store at 4 °C

14. Insulin-Transferrin-Selenium-Sodium Pyruvate (100×) (ITS-A) (ThermoFisher Scientific, catalog number: 51300044); store at 4 °C

15. 2-Mercaptoethanol (ThermoFisher Scientific, catalog number: 21985023); store at 4 °C

16. Penicillin-Streptomycin (10,000 U/mL) (Pen/Strep) (ThermoFisher Scientific, catalog number: 15140122); store at 4 °C

17. SB 431542 (Activin/BMP/TGF-β pathway inhibitor) (Tocris, catalog number: 1614)

**Critical:** It is required to check the lot number through tocris.com to confirm batch molecular weight and calculate dilution.

Reconstitute in DMSO to a concentration of 10 mM; store as aliquots at -20 °C.

18. LDN 193189 (Activin/BMP/TGF- β pathway inhibitor) (Tocris, catalog number: 6053)

**Critical**: It is required to check the lot number through tocris.com to confirm batch molecular weight and calculate dilution.

Reconstitute through two serial dilutions. First, reconstitute in DMSO to achieve a concentration of 25 mM stock solution. Further dilute 20 μL of the 25 mM stock solution with 980 μL of DMSO to achieve a concentration of 0.5 mM. Store aliquots at -20 °C.

19. Fibroblast growth factor-basic, human (bFGF) (Millipore Sigma, catalog number: F0291-25UG); reconstitute in 1 mL of sterile DH_2_O + 0.1% BSA to reach 25 mg/mL; store aliquots at -20 °C

20. Anti-adherence rinsing solution (STEMCELL Technologies, catalog number: 07010); store at RT

21. BrainPhys^TM^ Neuronal Medium N2-A & SM1 Kit (BrainPhys, SM1, N2-A) (STEMCELL Technologies, catalog number 05793); note that the kit comes with two supplements; store BrainPhys at 4 °C, and SM1 and N2-A at -20 °C

22. N6,2′-O-Dibutyryladenosine 3′,5′-cyclic monophosphate sodium salt (cAMP) (Millipore Sigma, catalog number: D0627-1G); reconstitute in 20 mL of dH_2_O to achieve a 50 mg/mL solution, and store aliquots at -20 °C

23. Ascorbic acid, 500 mg (STEMCELL Technologies, catalog number: 72132); reconstitute through two serial dilutions. First, reconstitute in 28.4 mL of H_2_O to achieve a concentration of 100 mM stock solution. Further dilute 10 μL of the 25 mM stock solution with 9.98 mL of H_2_O to achieve a concentration of 0.5 mM. Store aliquots at -20 °C

24. Brain-derived neurotrophic factor (BDNF) (Cedarlane Labs, catalog number: CLY100-01-100UG); reconstitute in 1 mL of dH_2_O + 0.1% BSA to achieve a 100 mg/mL solution, and store aliquots at -20 °C

25. Glial cell line-derived neurotrophic factor (GDNF) (Cedarlane Labs, catalog number: CLY100-02-100UG); reconstitute in 1 mL of dH_2_O + 0.1% BSA to achieve a 100 mg/mL solution, and store aliquots at -20 °C

26. Essential 8^TM^ Medium (E8) (ThermoFisher Scientific, catalog number: A1517001); see Recipe 5 for details

27. Human recombinant bone morphogenic protein 4 (BMP-4) (STEMCELL Technologies, catalog number: 78211); reconstitute in 40 μL of dH_2_O to achieve a 500 μg/mL solution and store aliquots at -20 °C

28. Recombinant human vascular endothelial growth factor 121 (VEGF-121) (BioLegend, catalog number: 583202); sold as 200 μg/mL, aliquot and store at -20 °C

29. Gibco stem cell factor (C Kit Ligand) recombinant human protein (SCF) (ThermoFisher Scientific, catalog number: PHC2115); reconstitute in 50 μL of dH_2_O BSA to achieve a 200 μg/mL solution, and store aliquots at -20 °C

30. X-VIVO^TM^ 15 serum-free hematopoietic cell medium, sterile, pH 6.9–7.2, with L-Glutamine, gentamicin, and phenol red, liquid (X-VIVO 15) (Lonza, catalog number: 04-418Q); store at 4 °C away from light

31. Human macrophage colony-stimulating factor recombinant protein (M-CSF) (ThermoFisher Scientific, catalog number: PHC9501); reconstitute in 100 μL of dH_2_O to achieve a 1 mg/mL solution, and store aliquots at -20 °C

32. Human recombinant IL-3 (IL-3) (STEMCELL Technologies, catalog number: 78040); reconstitute in 400 μL of dH_2_O to achieve a 250 μg/mL solution, and store aliquots at -20 °C

33. Advanced DMEM/F12 (ThermoFisher Scientific, catalog number: 12634010); store at 4 °C

34. Human recombinant granulocyte-macrophage colony-stimulating factor (*E. coli*-expressed) (GM-CSF) (STEMCELL Technologies, catalog number: 78015.1); reconstitute in 200 μL of dH_2_O to achieve a 100 μg/mL solution, and store aliquots at -20 °C

35. Recombinant human IL-34 (carrier-free) 100 μg (IL-34) (BioLegend, catalog number: 577906); sold at varying concentrations ~1 mg/mL. If above this concentration, adjust by adding dH_2_O to achieve 1 mg/mL and follow Recipe 7. If below, adjust Recipe 7 as needed to compensate

36. Recombinant human TGF-β1 (HEK293 derived) (TGF-β1) (PeproTech, catalog number: 100-21-250UG); reconstitute in 500 μL of dH_2_O to achieve a 500 μg/mL solution, and store aliquots at -20 °C

37. TrypLE^TM^ Express enzyme (1×), no phenol red (TrypLE) (ThermoFisher Scientific, catalog number: 12604039); store at RT

38. Versene solution (ThermoFisher Scientific, catalog number: 15040066); store at 4 °C

**Solutions**

1. Basal neural maintenance medium (BNMM) (see Recipes)

2. Neurosphere expansion medium (NSE) (see Recipes)

3. Neurosphere maturation medium (NSM) (see Recipes)

4. Neurosphere differentiation medium (NSD) (see Recipes)

5. Embryoid body medium (EBM) (see Recipes)

6. Hematopoietic differentiation medium (HDM) (see Recipes)

7. Microglial differentiation medium (MDM) (see Recipes)

**Recipes**

*Note: Many of the recipes listed below require additional supplements added fresh before use. If this is the case, supplements will be listed alongside the recipe name within the Procedure (e.g., BNMM + 10 μM Y-27632 means BNMM with Y-27632* ***added immediately before changing media*** *to reach a final concentration of 10 μM).*

**1. Basal neural maintenance medium (BNMM)**

**Table:** 

Reagent	Final concentration	Quantity or volume
DMEM/F12	n/a (base)	239.8 mL
Neurobasal	n/a (base)	239.8 mL
B-27	1%	5 mL
N-2	0.5%	2.5 mL
Pen/Strep	1% (100 IU)	5 mL
GlutaMAX	0.5%	2.5 mL
MEM-NEAA	0.5%	2.5 mL
ITS-A	0.5%	2.5 mL
2-mercaptoethanol	0.08%	400 μL
**Total**	n/a	500 mL

a. Thaw frozen supplements at room temperature (RT).

b. Filter-sterilize using a 500 mL vacuum filter bottle.

c. Keep media at 4 °C and use within 1 month of being mixed.

d. If a smaller volume is desired, media recipe can be reduced by 10× and filter-sterilized using a Steriflip^®^ vacuum tube top filter.

**2. Neurosphere expansion medium (NSE)**

**Table:** 

Reagent	Final concentration	Quantity or volume
DMEM/F12	n/a (base)	236.25 mL
Neurobasal	n/a (base)	236.25 mL
B-27	2%	10 mL
N-2	1%	5 mL
Pen/Strep	1% (100 IU)	5 mL
GlutaMAX	0.5%	2.5 mL
MEM-NEAA	0.5%	2.5 mL
ITS-A	0.5%	2.5 mL
**Total**	n/a	500 mL

a. Thaw frozen supplements at RT. Filter-sterilize using a 500 mL vacuum filter bottle.

b. Keep media at 4 °C and use within 1 month of being mixed.

c. If a smaller volume is desired, the recipe can be reduced by 10× and filter-sterilized using a Steriflip^®^ vacuum tube top filter.

**3. Neurosphere maturation medium (NSM)**

**Table:** 

Reagent	Final concentration	Quantity or volume
BrainPhys	n/a (base)	480 mL
SM1	2%	10 mL
N2-A	1%	5 mL
Pen/Strep	1% (100 IU)	5 mL
**Total**	n/a	500 mL

a. Thaw frozen supplements at RT. Filter-sterilize using a 500 mL vacuum filter bottle.

b. Keep media at 4 °C and use within 1 month of being mixed.

c. If a smaller volume is desired, the recipe can be reduced by 10× and filter-sterilized using a Steriflip^®^ vacuum tube top filter.

**4. Neurosphere differentiation medium (NSD)**

**Table:** 

Reagent	Final concentration	Quantity or volume
BrainPhys	n/a (base)	484 mL
SM1	2%	10 mL
N2-A	1%	5 mL
Pen/Strep	1% (100 IU)	5 mL
cAMP (50 mg/mL)	500 μg/mL	5 mL
Ascorbic acid (100 μM)	200 nM	1 mL
**Total**	n/a	500 mL

a. Thaw frozen supplements at RT. Filter-sterilize using a 500 mL vacuum filter bottle.

b. Keep media at 4 °C and use within 1 month of being mixed.

c. If a smaller volume is desired, the recipe can be reduced by 10× and filter-sterilized using a Steriflip^®^ vacuum tube top filter.

d. Note that NSD only differs from NSM in cAMP and ascorbic acid, so an alternative strategy is to add these supplements to already mixed NSM at the listed ratios.

**5. Embryoid body medium (EBM)**

**Table:** 

Reagent	Final concentration	Quantity or volume
E8 base media	n/a (base)	9.79 mL
E8 Supplement	2%	0.2 mL
BMP4 (500 μg/mL)	50 ng/mL	1 μL
SCF (200 μg/mL)	20 ng/mL	1 μL
VEGF-121 (200 μg/mL)	50 ng/mL	2.5 μL
Y-27632 (10 mM)	10 μM	10 μL
**Total**		10 mL

**Critical**: E8 medium is provided as a base medium (500 mL) and supplement (10 mL). We have found that it is necessary to immediately freeze both the base medium in 50 mL aliquots and the supplement in 1 mL aliquots at -20 °C. Thaw all components at RT and use within 1 week of mixing.

**6. Hematopoietic differentiation medium (HDM)**

**Table:** 

Reagent	Final concentration	Quantity or volume
X-VIVO 15	n/a (base)	48.94 mL
GlutaMAX	1%	0.5 mL
Pen/Strep	1% (100 IU)	0.5 mL
2-mercaptoethanol	0.1%	50 μL
M-CSF (1 mg/mL)	100 ng/mL	5 μL
IL-3 (250 μg/mL)	25 ng/mL	5 μL
**Total**		50 mL

Thaw frozen supplements at RT. Keep media at 4 °C and use within 1 month of being mixed.

**7. Microglial differentiation medium (MDM)**

**Table:** 

Reagent	Final concentration	Quantity or volume
Advanced DMEM/F12	n/a (base)	48.98 mL
GlutaMAX	1%	0.5 mL
Pen/Strep	1%	0.5 mL
GM-CSF (100 μg/mL)	10 ng/mL	5 μL
IL-34 (1 mg/mL)	100 ng/mL	5 μL
TGFβ1 (500 μg/mL)	50 ng/mL	5 μL
M-CSF (1 mg/mL)	25 ng/mL	1.25 μL
**Total**		50 mL

Thaw frozen supplements at RT. Keep media at 4 °C and use within 2 weeks of being mixed.

**Laboratory supplies**

1. Corning^®^ Isotip^®^ filtered pipette tips, 0.1–2 µL (Millipore Sigma, catalog number: CLS4801-960EA)

2. Axygen 20 μL maximum recovery universal fit filter tips racked, clear, sterile, rack pack (VWR, catalog number: 22234-008)

3. 200 μL filter tips retention, sterile rack, 96 tips/rack, 10 racks/pack (ESBE Scientific, catalog number: ESB-VAR200200PT)

4. 1,000 μL filter tips retention, sterile rack, 96 tips/rack, 10 racks/pack (ESBE Scientific, catalog number: ESB-VAR201000PT)

5. Optifit P1000 wide-bore tips, Sartorius (VWR, catalog number: CA15000-468)

6. Thermo Scientific ClipTip^TM^ 1250 µL filtered pipette tips (ThermoFisher Scientific, catalog number: 94420813)

7. Nunc^TM^ serological pipettes, 5 mL (Tapered tip) (Thermo Scientific, catalog number: 170355N)

8. Nunc^TM^ serological pipettes, 10 mL (Tapered tip) (Thermo Scientific, catalog number: 170356N)

9. Thermo Scientific^TM^ Nunc^TM^ serological pipettes, 25 mL (Thermo Fisher Scientific, catalog number: 170357N)

10. Falcon^®^ 6-well polystyrene microplate (ThermoFisher Scientific, catalog number: 08-772-33)

11. Falcon^®^ 15 mL conical bottom centrifuge tube (VWR, catalog number: CA21008-918)

12. Falcon^®^ 50 mL conical bottom centrifuge tube (VWR, catalog number: CA21008-951)

13. Vacuum filtration systems 500 mL (VWR, catalog number: 10040-436)

14. Steriflip-GV, 0.22 μm, PVDF, radio-sterilized (EMD Millipore, catalog number: SE1M179M6)

15. Mr. Frosty^TM^ freezing container (ThermoFisher Scientific, catalog number: 5100-0001)

16. AggreWell^TM^ 800 Microwell 24-well culture plates (STEMCELL Technologies, catalog number: 34815)

17. Large 37 μm reversible strainer (STEMCELL Technologies, catalog number: 27250)

18. Nunc^TM^ EasYFlask^TM^ 75 cm^2^ polystyrene tissue culture treated flasks, sterile, filter cap, angled neck (ThermoFisher Scientific, catalog number: 156499)

19. Cellvis 6-well glass bottom plate with high performance #1.5 cover glass, 20 pack (ThermoFisher Scientific, catalog number: NC0452316)

20. Vwr^®^ tissue culture dish, non-treated, sterilized, non-pyrogenic, diameter 9.0 cm, growth area 55 cm^2^ (VWR, catalog number: 10861-592)

21. VWR^®^ disposable pipetting reservoirs (sterile) (VWR, catalog number: 89108-002)

22. Falcon^®^ cell scrapers, sterile (Corning, catalog number: CA15621-005)

## Equipment

*Note: Only specific, more specialized cell culture equipment that may not be found in every cell culture lab space is listed below. Basic cell culture equipment (BSC hood, incubator, etc.) is required but omitted from the following list.*

1. Incubator

2. Tissue culture hood

3. Light microscope

4. Dissection microscope (ZEISS, model: SteREO Discovery.V8 or RWD Life Science, DOM 1001)

5. Gilson Pipetman L 4-Pack Starter Kit, P2L, P20L, P200L, P1000L (Mandel, catalog number: GF-F167370)

6. Pipet Aid^®^ Portable pipetting device, 110 V, Drummond Scientific 4-000-100, 1/EA (ThermoFisher Scientific, catalog number: 13-681-19)

7. E1-ClipTip; Bluetooth; electronic adjustable tip spacing multichannel equalizer pipettes (ThermoFisher Scientific, catalog number: 4672090BT)

8. Celltron orbital shaker system (Infors HT)

## Procedure

**A. iPSC to NPC differentiation**

1. Thaw iPSCs onto wells of a Matrigel-coated 6-well plate at a density of approximately 5 × 10^5^ cells/well.

a. Coat a 6-well plate with Matrigel according to the manufacturer’s guidelines with a 2-h incubation at 37 °C with 5% CO_2_.

b. Gently thaw iPSCs at RT. Just as the iPSCs fully thaw, transfer to a 15 mL conical centrifuge tube and pipette 7 mL of DMEM/F12 dropwise.

c. Centrifuge at 300× *g* for 5 min.

d. Discard the supernatant, taking care not to disturb the cell pellet.

e. Resuspend in mTeSR^TM^ Plus + 10 μM Y-27632 (ROCK inhibitor to enhance cell survival) at a volume of 2 mL/5 × 10^5^ cells.

f. Plate 2 mL/well onto the Matrigel-coated 6-well plate.

g. Incubate at 37 °C with 5% CO_2_.

2. Twelve hours after plating, perform 100% media change to fresh mTeSR^TM^ Plus **without** Y-27632. Continue performing 100% media changes every 24 h until cells reach 95% confluence.

*Note: The remainder of section A is written as though a single cell line/differentiation is being produced. Adjust as necessary for multiple cell lines/separate differentiations.*

3. When cells reach 95% confluence, detach with Accutase and passage onto one well of a new Matrigel-coated 6-well plate at a ratio of 2:1 (i.e., passage two 95% confluent wells onto one new well).

a. Coat a single well of a 6-well plate with Matrigel as described above.

b. Remove media from one well of cells, wash once with PBS, and add 1 mL of Accutase that has been prewarmed for 5 min in a 37 °C hot water bath.

c. Incubate for 5–10 min at 37 °C with 5% CO_2_.

d. Once cells are detached, deactivate Accutase by adding 3–4 mL of DMEM/F12 and transfer to a 15 mL conical centrifuge tube.

e. Centrifuge at 300× *g* for 5 min.

f. Discard supernatant, taking care not to disturb the cell pellet.

g. Resuspend the entire pellet in 2 mL of mTeSR^TM^ Plus + 10 μM Y-27632 until a single-cell condition and transfer onto the single-well of the Matrigel-coated 6-well plate.

h. Incubate at 37 °C with 5% CO_2_ for 24 h.

4. Twenty-four hours later, observe that cells are 95%–100% confluent. At this point, cells are considered DIV 1. Perform 100% media change to BNMM + 10 μM SB 431542 and 0.5 μM LDN 193189 (Activin/BMP/TGF-β pathway inhibitors that promote differentiation of iPSCs to NPCs) + 10 μM Y-27632.

*Note: It is critical that cells are 100% confluent 24 h after plating. You may find that this requires slightly altering the 2:1 ratio in step A3 to achieve this.*

5. Continue with daily 100% media changes to BNMM + 10 μM SB 431542 and 0.5 μM LDN 193189 + 10 μM Y-27632 until DIV 8.

6. On DIV 8, detach cells with Versene and passage onto a new Matrigel-coated 6-well plate at a ratio of 1:3.

a. Coat three wells of a 6-well plate with Matrigel as described above.

b. Remove media from one well of cells, wash once with PBS, and add 1 mL of Versene that has been prewarmed for 5 min in a 37 °C hot water bath.

c. Incubate for 5–10 min at 37 °C with 5% CO_2_.

d. Gently aspirate Versene while preserving the cell layer.

e. Add 3 mL of BNMM + 10 μM SB 431542 and 0.5 μM LDN 193189 + 10 μM Y-27632. Gently swirl using a cell scraper and/or pipette the cells 2–3 times slowly in order to maintain aggregates of 50–100 cells.

f. Transfer 1 mL/well of the cells onto the Matrigel-coated plate. Top up the media with an additional 1 mL of BNMM + 10 μM SB 431542 and 0.5 μM LDN 193189 + 10 μM Y-27632/well.

g. Incubate at 37 °C with 5% CO_2_ for 24 h.

**Figure 1.**
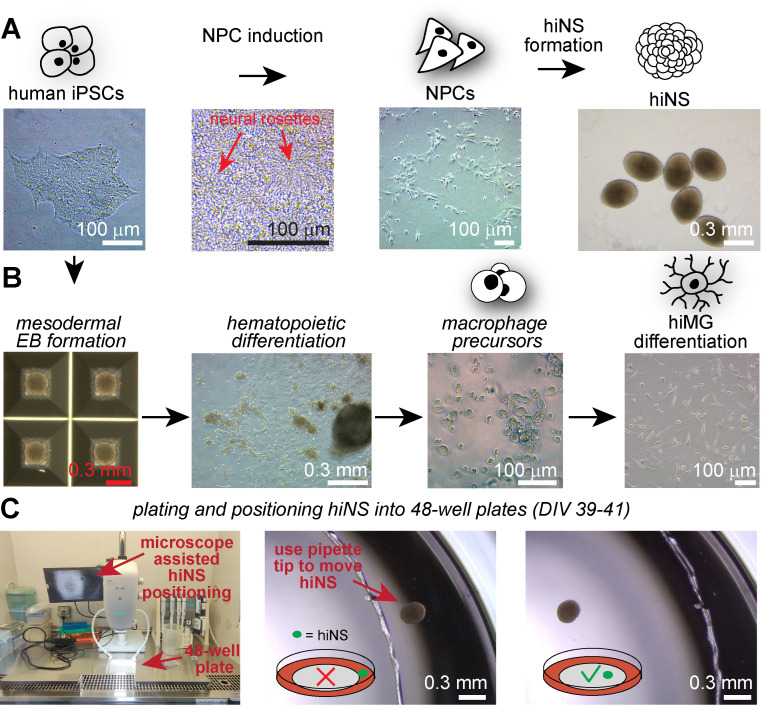
Example microscopy images of all induced pluripotent stem cells (iPSCs)-derived cell culture stages necessary for human iPSC-derived 3D neurosphere (hiNS) generation. (A) Human iPSC colonies are used for dual SMAD inhibition–based neural precursor cell (NPC) induction. Note the dense monolayer during NPC induction and the presence of rosette-like structures. NPCs can then be frozen down and thawed prior to induction of the hiNS protocol. **(B)** Parallel differentiation from iPSCs to mature human iPSC-derived microglia (hiMG). iPSCs are initially differentiated into mesodermal embryoid bodies (EBs) using AggreWell plates, followed by a hematopoietic differentiation period in T75 cell culture flasks in which macrophage precursors accumulate. Free-floating macrophage precursors can be harvested and differentiated into hiMG, prior to their addition to hiNS. **(C)** Between DIV 39 and 41, hiNS are plated into 48-well plates with the help of a tissue dissection microscope (RWD DOM-1001) within the cell culture cabinet. After transferring hiNS with a wide bore tip, carefully guide hiNS with a small pipette tip to the desired location for attachment. hiNS will attach within 24 h on Matrigel-coated tissue culture surfaces.

7. On DIV 9, perform a 100% media change to BNMM with no additional supplements. Continue with daily 100% media changes with BNMM until DIV 12.

8. On DIV 12, observe neural rosette structure (see Figure 1A for expected neural rosette morphologies, indicated by arrows) and perform 100% media change to BNMM + 20 ng/mL bFGF (sustains NPC proliferation); 48 h later, on DIV 14, perform another 100% media change to BNMM + 20 ng/mL bFGF.

9. On DIV 16, detach cells with Accutase and passage onto new Matrigel-coated 6-well plates at a ratio of 1:3.

a. Coat 9 wells of two 6-well plates with Matrigel as described above.

b. Remove media from three wells of cells, wash once with PBS, and add 1 mL/well of Accutase that has been prewarmed for 5 min in a 37 °C hot water bath.

c. Incubate for 5–10 min at 37 °C with 5% CO_2_.

d. Once cells are detached, deactivate Accutase by adding 3–4 mL of DMEM/F12 to each well, and transfer to a 15 mL conical centrifuge tube.

e. Centrifuge at 300× *g* for 5 min.

f. Discard the supernatant, taking care not to disturb the cell pellet.

g. Resuspend the entire pellet in 9 mL of BNMM + 20 ng/mL bFGF to the single-cell condition.

h. Transfer 1 mL/well of the cells onto the Matrigel-coated plate. Top up the media with an additional 1 mL of BNMM + 20 ng/mL bFGF.

i. Incubate at 37 °C with 5% CO_2_ for 24 h.

10. On DIV 17, perform 100% media change to BNMM + 20 ng/mL bFGF. Continue performing 100% media changes to BNMM + 20 ng/mL bFGF every 48 h until DIV 23.

11. On DIV 23, cells have completed differentiation into NPCs; detach cells with Accutase and freeze down at 20 × 10^6^ cells/cryovial.

a. Remove media from the 9 wells of cells, wash once with PBS, and add 1 mL/well of Accutase that has been prewarmed for 5 min in a 37 °C hot water bath.

b. Incubate for 5–10 min at 37 °C with 5% CO_2_.

c. Once cells are detached, deactivate Accutase by adding 3–4 mL of DMEM/F12 to each well and transfer to a 50 mL conical centrifuge tube.

d. Centrifuge at 300× *g* for 5 min.

e. Discard the supernatant, taking care not to disturb the cell pellet.

f. Resuspend the cells in 10 mL of BNMM + 10% DMSO + 20 ng/mL bFGF + 10 μM Y-27632 to the single-cell condition.

g. Count cells and adjust media volume as necessary to reach 20 × 10^6^ cells/mL.

h. Transfer 1 mL into each cryovial and freeze cells down for 24 h at -80 °C using a Mr. Frosty^TM^ freezing container.

i. Twenty-four hours later, transfer cells to liquid nitrogen for long-term storage.

**Pause point:** At this point, cells are labeled as passage 0 (P0) NPCs and can be used for neurosphere differentiation or further expanded to increase cell number. In our protocol, we have successfully differentiated neurospheres from P1 to P5 NPCs, with the majority of neurosphere differentiation being performed on P2 NPCs (see Figure 1 for expected NPC morphologies). The steps that follow will describe the expansion of NPCs from P0 to P2.

12. Thaw one vial of P0 NPCs onto a full Matrigel-coated 6-well plate at a density of approximately 20 × 10^6^ cells/plate.

a. Coat a 6-well plate with Matrigel according to the manufacturer’s guidelines, with a 2-h incubation.

b. Gently thaw NPCs at RT. Just as the NPCs fully thaw, transfer to a 15 mL conical centrifuge tube and pipette 7 mL of DMEM/F12 dropwise.

c. Centrifuge at 300× *g* for 5 min.

d. Discard the supernatant, taking care not to disturb the cell pellet.

e. Resuspend in 12 mL of NSE + 20 ng/mL bFGF + 10 μM Y-27632.

f. Plate 2 mL/well onto the Matrigel-coated 6-well plate.

g. Incubate at 37 °C with 5% CO_2_.

13. Twelve hours following plating, perform a 100% media change to fresh NSE + 20 ng/mL bFGF **without** Y-27632. Continue performing 100% media changes every 24 h for 1 week.

*Note: At this point, the number of wells and the total media requirements may vary depending on how many cells you wish to expand. Numbers will be written more generally for the following steps.*

14. One week later, detach cells with Accutase and passage NPCs to a new Matrigel-coated 6-well plate at a ratio of 1:3.

a. Coat as many 6-well plates as necessary for a 1:3 split with Matrigel as described above.

b. Remove media from the wells of NPCs, wash once with PBS, and add 1 mL/well of Accutase that has been prewarmed for 5 min in a 37 °C hot water bath.

c. Incubate for 5–10 min at 37 °C with 5% CO_2_.

d. Once cells are detached, deactivate Accutase by adding 3–4 mL of DMEM/F12 to each well, and transfer to a 15 or 50 mL conical centrifuge tube.

e. Centrifuge at 300× *g* for 5 min.

f. Discard the supernatant, taking care not to disturb the cell pellet.

g. Resuspend the entire pellet in BNMM + 20 ng/mL bFGF + 10 μM Y-27632 at a volume of 1 mL per each new well to be plated onto.

h. Transfer 1 mL/well of the cells onto the Matrigel-coated plate. Top up the media with an additional 1 mL of BNMM + 20 ng/mL bFGF.

i. Incubate at 37 °C with 5% CO_2_ for 24 h.

15. Twelve hours following plating, perform a 100% media change to fresh NSE + 20 ng/mL bFGF **without** Y-27632. Continue performing 100% media changes every 24 h for ~1 week.

16. Approximately 1 week later, detach cells with Accutase and freeze down cells at 10 × 10^6^ cells/cryovial. See detailed description in step A11, adjusting for the number of wells. Label cells at this point as P2 NPCs.

**B. Microglia differentiation**

The protocol presented here for human iPSC-derived microglia differentiation was adapted from the Cowley lab [15].

1. Thaw iPSCs onto wells of a Matrigel-coated 6-well plate at a density of approximately 5 × 10^5^ cells/well.

a. See detailed description in step A1.

2. Twelve hours after plating, perform a 100% media change to fresh mTeSR^TM^ Plus **without** Y-27632. Continue performing 100% media changes every 24 h until cells reach 80%–90% confluence.

*Note: The remainder of section B is written as though a single cell line/differentiation is being produced. Adjust as necessary for multiple cell lines/separate differentiations.*

3. When cells reach 80%–90% confluence, detach cells with Accutase and passage 1.5 × 10^6^ cells onto a single well of an AggreWell 800 cell culture plate.

a. Prepare a single well of the AggreWell 800 plate. Add 1 mL of anti-adherence rinsing solution and centrifuge the plate at 1,000× *g* for 3 min. Remove anti-adherence solution, rinse the well once with 2 mL of DMEM, and add 1 mL of EBM.

b. Remove all media from each well of the iPSCs, wash once with PBS, and add 1 mL/well of Accutase that has been prewarmed for 5 min in a 37 °C hot water bath.

c. Incubate for 5–10 min at 37 °C with 5% CO_2_.

d. Once cells are detached, deactivate Accutase by adding 3–4 mL of DMEM/F12 to each well and transfer to a 15- or 50-mL conical centrifuge tube (depending on the number of wells).

e. Centrifuge at 300× *g* for 5 min.

f. Discard the supernatant, taking care not to disturb the cell pellet.

g. Resuspend the cells in 10 mL of EBM and count.

h. Transfer 1.0–1.5 × 10^6^ iPSCs onto the single well of the AggreWell plate.

*Note: We found that using 1–1.2 × 10^6^ iPSCs can result in EBs with more consistent morphologies, depending on cell line.*

i. Centrifuge at 100× *g* for 5 min.

j. Incubate at 37 °C with 5% CO_2_ for 24 h.

4. Twenty-four hours later, note embryoid body (EB) formation. Cells are considered DIV 1. Perform a 100% media change to EBM. Continue performing 100% media changes every 24 h until DIV 5.

5. On DIV 5, transfer EBs onto an uncoated 75 mm flask in HDM.

a. Using a p1000 wide-bore tip, gently pipette up and down inside the AggreWell to dislodge the EBs and transfer to a reversible strainer that has been placed upon an empty 50 mL conical centrifuge tube. Pipette an additional 1 mL of DMEM into the AggreWell and then transfer to the strainer to dislodge any remaining EBs.

*Note: The 50 mL conical centrifuge tube will contain single cells/debris, while the strainer will contain the EBs.*

b. Place a new 50 mL conical centrifuge tube upside-down on the top of the strainer (i.e., the side of the strainer upon which the EBs are placed), reverse the strainer and tube so that they are right-side up, and run 11 mL of HDM through the strainer to dislodge the EBs fully into the tube.

c. Transfer the HDM and EBs onto an uncoated 75 mm flask.

d. Incubate at 37 °C with 5% CO_2_ for ~1 week.

**Critical:** Ensure that the flask does not move once placed for 1 week to allow for maximum attachment. We suggest placing it in the very back of the incubator to avoid any accidental movement.

6. Approximately 1 week later, observe attachment of the EBs onto the flask. The percentage of EBs attaching to the bottom of the flask can vary from batch to batch. Some EBs should attach and look similar to the example image in Figure 1B. However, if a fraction of EBs remain free-floating, it is not a problem in our experience. The flask is termed “factory”.

**Pause point:** From here until ~6 months, perform twice-weekly medium top-ups with 5 mL of HDM. EBs will consistently produce macrophage-like cells and can be regularly harvested starting from 1-month post-plating for microglial differentiation. The following steps in the protocol can be performed at any point between 1 and 6 months on harvested macrophages. We have found that it is ideal to keep the factory media level below 30 mL.

7. Collect macrophages from the factory and plate onto a glass-bottom 6-well plate at a density of 5 × 10^5^–1 × 10^6^ per well.

a. Collect the desired amount of media from the factory and run the media through a reversible strainer placed upon an empty 50 mL conical tube.

*Note: The 50 mL conical tube will contain macrophage-like cells, while the strainer will contain any floating EBs that may have been caught during harvesting.*

**Critical**: Any floating EBs must be returned to the factory, or the factory risks collapse.

b. Place a new 50 mL conical centrifuge tube upside-down on the top of the strainer (i.e., the side of the strainer upon which the EBs are placed), reverse the strainer and tube so that they are right-side up, and run 5 mL of HDM through the strainer to dislodge the EBs fully into the tube.

c. Transfer the HDM and EBs back to the factory.

d. To the original 50 mL conical centrifuge tube that contains the macrophage-like cells, centrifuge at 300× *g* for 5 min.

e. Discard the supernatant, taking care not to disturb the cell pellet.

f. Resuspend the cells in 1 mL of MDM.

g. Count cells and adjust the MDM volume as necessary to reach 3 mL of media for every 5 × 10^5^–1 × 10^6^ of cells.

h. Transfer 3 mL of cells in MDM into each well of an uncoated glass-bottom 6-well plate.

i. Incubate at 37 °C with 5% CO_2_ for 24 h.

8. Twenty-four hours later, note attachment of cells and perform a 50% media change. Continue performing 50% media changes daily for the next 7–10 days.

9. After 7–10 days of differentiation, cells are considered microglia and are ready to be added to neurospheres (see step D4).

**C. Neurosphere formation, differentiation, and maturation until plating**

1. Thaw P1–P3 NPCs onto a full Matrigel-coated 6-well plate at a density of approximately 2–3 × 10^6^ cells/plate.

a. See detailed description in step A12.

2. Twelve hours following plating, perform a 100% media change to fresh NSE + 20 ng/mL bFGF **without** Y-27632. Continue performing 100% media changes every 24 h for ~1 week.

*Note: The remainder of section C is written as though a single cell line/differentiation is being produced. Adjust as necessary for multiple cell lines/separate differentiations.*

3. After ~1 week, detach cells with Accutase and passage 1.2 × 10^6^ cells onto a single well of an AggreWell 800 cell culture plate.

a. Prepare a single well of the AggreWell 800 plate. Add 1 mL of anti-adherence rinsing solution and centrifuge the plate at 1,000× *g* for 3 min. Remove anti-adherence solution, rinse the well once with 2 mL of DMEM, and add 1 mL of NSE + 20 ng/mL bFGF + 10 μM Y-27632.

b. Remove all media from one well of the NPCs, wash once with PBS, and add 1 mL of Accutase that has been prewarmed for 5 min in a 37 °C hot water bath.

*Note: The required number of NPCs per AggreWell (1.2 × 10^6^) is far below the original plated number of NPCs in step C1. This is to keep consistency with our own lab culture standards for NPCs. Likely, only one well of the 6-well plate is necessary to reach this cell number; adjust if necessary.*

c. Incubate for 5–10 min at 37 °C with 5% CO_2_.

d. Once cells are detached, deactivate Accutase by adding 3–4 mL of DMEM/F12 and transfer to a 15 mL conical centrifuge tube.

e. Centrifuge at 300× *g* for 5 min.

f. Discard supernatant, taking care not to disturb the cell pellet.

g. Resuspend the cells in 1 mL of NSE + 20 ng/mL bFGF + 10 μM Y-27632 and count.

h. Transfer 1.2 × 10^6^ NPCs onto the single well of the AggreWell plate.

*Note: AggreWell 800 24-well plates contain ~300 microwells per well. Our optimal neurosphere formation size is 4,000 cells/well, hence 1.2 × 10^6^ cells. Changing this number may be desired if working with different cell lines.*

i. Centrifuge at 100× *g* for 5 min.

j. Incubate at 37 °C with 5% CO_2_ for 24 h.

4. Twenty-four hours later, note neurosphere formation. Cells are considered DIV 1. Perform a 100% media change to 1.5 mL of NSE + 20 ng/mL bFGF **without** Y-27632. Continue performing 100% media changes every 24 h until DIV 7.

**Critical**: It is vitally important that media is removed and added extremely slowly, as going too fast will dislodge the neurospheres, causing them to fuse. Use a P1000 pipette, preferably one that moves very smoothly, and try to pipette as slowly as possible. It can take up to a minute per 750 μL pipetting step in order to safely change media. You should check on the wells after each media change to ensure you are not dislodging neurospheres. Speed up or slow down to find an optimal speed for your pipette.

5. At DIV 7, transfer neurospheres to an uncoated 9 cm Petri dish and incubate on an orbital shaker plate at 65 rpm.

a. Using a p1000 wide-bore tip, gently pipette up and down inside the AggreWell to dislodge the neurospheres and transfer to a reversible strainer that has been placed upon an empty 50 mL conical centrifuge tube. Pipette an additional 1 mL of DMEM into the AggreWell and then transfer to the strainer to dislodge any remaining neurospheres.

*Note: The 50 mL conical centrifuge tube will contain single cells/debris, while the strainer will contain the neurospheres.*

b. Place a new 50 mL conical centrifuge tube upside-down on the top of the strainer (i.e., the side of the strainer upon which the NPCs are placed), reverse the strainer and tube so that they are right-side up, and run 11 mL of NSD + 20 ng/mL of BDNF and GDNF through the strainer to dislodge the neurospheres fully into the tube.

c. Transfer the NSD and neurospheres onto an uncoated 9 cm cell culture dish.

d. Incubate on a shaker plate set to 65 rpm (using a Celltron orbital shaker) at 37 °C with 5% CO_2_ for 2 days.

6. On DIV 9, perform a 100% media change to 10 mL of NSD + 20 ng/mL of BDNF and GDNF (promoters of neuronal differentiation).

a. To change media, swirl the dish until the spheres are collected together, then gently tilt the plate to allow the neurospheres to collect on one side. Carefully aspirate as much media as possible without aspirating or damaging the neurospheres.

b. Add 10 mL of NSD + 20 ng/mL of BDNF and GDNF.

7. Continue to perform 100% media changes every 2–3 days until DIV 28.

*Note: It is likely that large amounts of cellular debris will appear in the well and can easily be confused for contamination. If uncertain, perform appropriate tests to rule out contamination.*

8. On DIV 28, perform a 100% media change to NSM + 20 ng/mL of BDNF and GDNF; continue to perform 100% media change every 2–3 days until neurospheres are plated (see section D).

**D. Neurosphere plating, full maturation, and analysis**

*Note: Further experimentation and analysis of the neurospheres will very often require that the neurospheres are both 1) attached to a plate, and 2) segregated into separate wells. This section describes the protocol for achieving these goals. It is feasible that neurospheres can continue in—and be experimented on—suspension culture far longer than our provided timeline, but this has not been tested by our group.*

1. At DIV 40, neurospheres are individually transferred and adhered to Matrigel-coated 24- or 48-well plates.

*Note: DIV 40 is a standard for our protocol, but the precise DIV for plating can vary depending on experimentation intentions.*

**Critical:** *Neurospheres will continue to appear healthy with high levels of electrical activity for a minimum of 3 weeks post-plating.*

a. Coat the desired number of wells of 24- or 48-well plates with Matrigel as described above using a minimum 1-h incubation.

*Note: We have successfully plated neurospheres onto Matrigel-coated standard cell culture plates, coverslips on a 24- or 48-well plate, microelectrode array (MEA) plates, and live-imaging glass-bottom plates.*

b. If not already present, transfer a dissection microscope into the cell culture biosafety cabinet, ensuring that all surfaces of the microscope and biosafety cabinet have been thoroughly cleaned with 70% ethanol.

c. Once the Matrigel coating is complete, pre-load all wells with 400 μL of NSM + 20 ng/mL of BDNF and GDNF.

*Note: 400 μL is used regardless of whether the plates have 24 or 48 wells.*

d. Using a P1000 wide-bore pipette tip, individually transfer neurospheres into single-wells until you have fully filled one plate.

e. At this point, you have the option of precisely positioning the neurospheres if necessary, such as positioning the neurospheres on top of a glass coverslip or directly over MEA plate electrodes. To position, view the individual well and neurosphere under the dissection microscope while using a small P20 pipette tip, P200 pipette tip, or blunt needle to gently nudge the neurospheres to the desired location.

f. Once neurospheres are positioned, incubate the cells at 37 °C with 5% CO_2_ for 24 h.

**Critical:** Be very gentle when transferring the plate to the incubator post-positioning so as not to dislodge the neurospheres.

**Figure 2.**
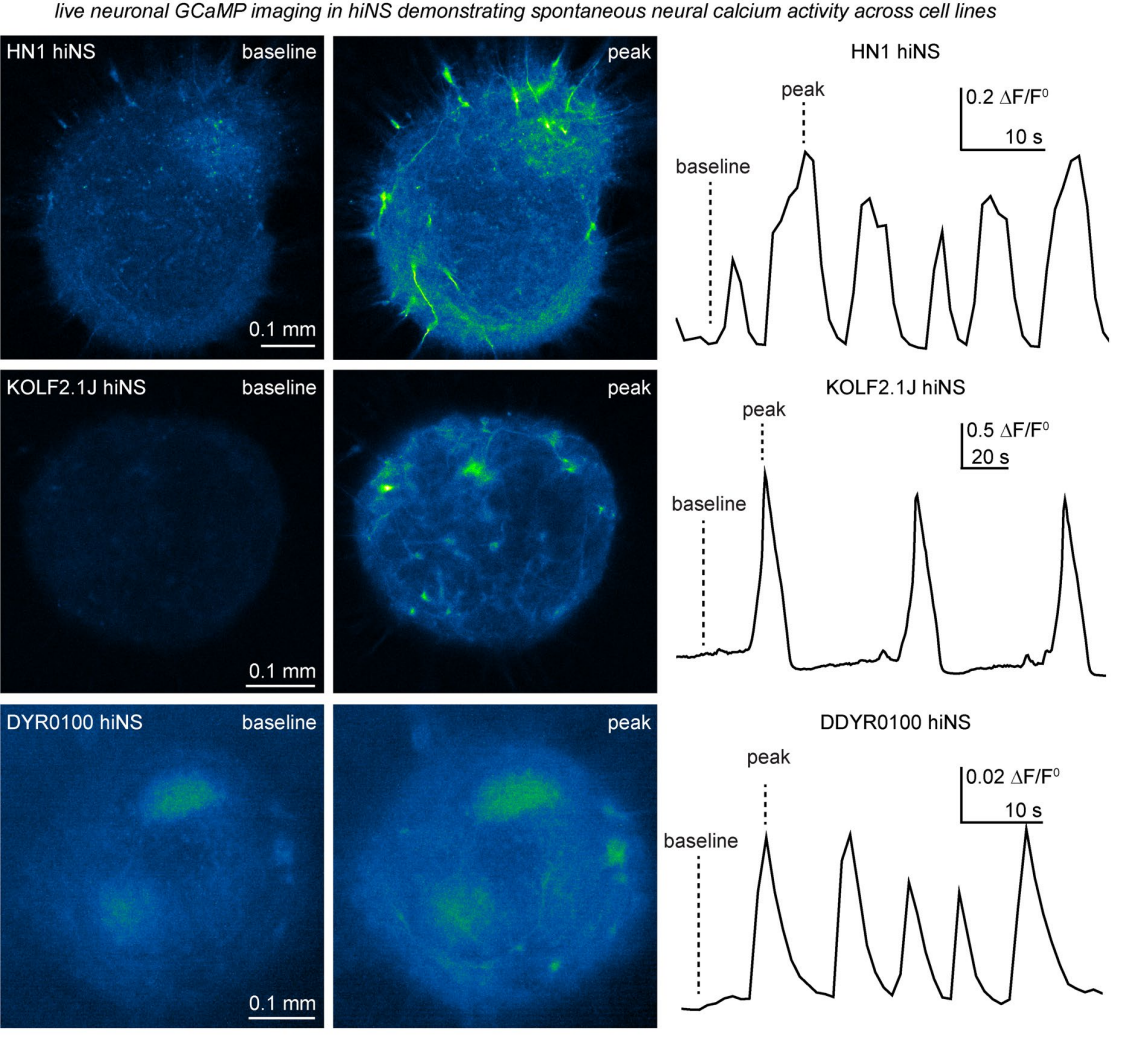
Demonstration of spontaneous neural calcium activity in human induced pluripotent stem cell (iPSC)-derived 3D neurospheres (hiNS) detected through hSyn-GCaMP6f expression and live confocal and epifluorescence microscopy. Our hiNS differentiation protocol results in highly functional, spontaneously active, 3D neural tissue. During or after plating hiNS onto Matrigel-coated glass bottom plates, adeno-associated virus (AAVs) were added to transduce hiNS [AAV.php.eb.-(HN1 and KOLF2.1J) or AAV2/9 (DYR0100)-hSyn-GCaMP6f, 0.5 mL/hiNS, titer: 1–2 E13]. Robust expression can be observed around 2 weeks post-transduction. The example presented here shows traces of hiNS derived from our in-house HN1 iPSC line, as well as the commercially available KOLF2.1J line, that were imaged using an LSM880 Zeiss confocal microscope with an attached live imaging incubator system at the University of British Columbia. In contrast, hiNS derived from the DDYR0100 iPSC lines were generated at the Shenzhen Institutes of Advanced Technology. Note the intrinsic differences in frequency and amplitudes of spontaneous activity. All recordings were done at 37 °C in a 5% CO_2_ atmosphere. The left side of the figure illustrates raw immunofluorescence signals of both baseline and peak activity in hiNS. The right side displays the calculated DF/F0 signal across the area of hiNS, illustrating the variety and differences of intrinsic calcium wave activities in hiNS derived from different iPSC lines.

2. After 24 h, observe attachment of neurospheres to wells. At this point, you may transduce neurospheres with adeno-associated virus (AAVs) if desired. We found AAVs to efficiently transduce neurons (using the hSyn promoter) and astrocytes (using the gfaABC1D promoter), allowing researchers to easily express genetically encoded sensors to perform functional imaging. Here, we show and demonstrate the utilization of hSyn-GCamp6f to monitor neuronal calcium activity in hiNS from three different iPSC lines (Figure 2).

a. For AAV transduction, we have utilized AAV.php.eb serotypes with a 0.5 mL/hiNS (titer: 1–2 E13) volume, e.g., add 24 µL of AAV to one full 48-well plate of neurospheres. Add AAV in a single media change as described in the following step.

3. Twenty-four to forty-eight hours after first plating, perform a ~37% media change on neurospheres by replacing 150 mL of media from each well with NSM + 20 ng/mL of BDNF and GDNF. Continue to perform ~37% media changes every 48–72 h until DIV 50.

*Note: Although it can be done without, we strongly recommend using an automatic multichannel pipette for media changes.*

a. With a multichannel pipette speed set to slow, remove 120 mL of media from each well.

*Note: This lower volume accounts for 20–30 mL of media evaporation that occurs every ~2 days.*

b. With a multichannel pipette speed set to slow, add 150 mL of NSM + 20 ng/mL of BDNF and GDNF (+AAV if required) to each well.

c. Incubate at 37 °C with 5% CO_2_. Repeat ~37% media changes every 48–72 h until DIV 50.

**Critical**: You **do not** need to add any more AAV to any further media changes.

4. On DIV 50, add microglia at a rate of 3 × 10^4^ microglia/neurosphere. Continue to culture until DIV 60.

a. Following step B9, mature microglia are ready to be added.

b. **To the microglia plate**, add 1 mL/well of prewarmed TrypLE onto each well of microglia. Incubate at 37 °C with 5% CO_2_ on an orbital shaker plate set to 65 rpm for 5–10 min.

c. Once cells are detached, deactivate TrypLE by adding 3–4 mL of DMEM/F12 and transfer to a 15 mL conical centrifuge tube.

d. Centrifuge at 300× *g* for 5 min.

e. Discard the supernatant, taking care not to disturb the cell pellet.

f. Resuspend the entire pellet in 1 mL of NSM + 20 ng/mL of BDNF and GDNF + IL-34/TGF-b and count microglia.

**Figure 3.**
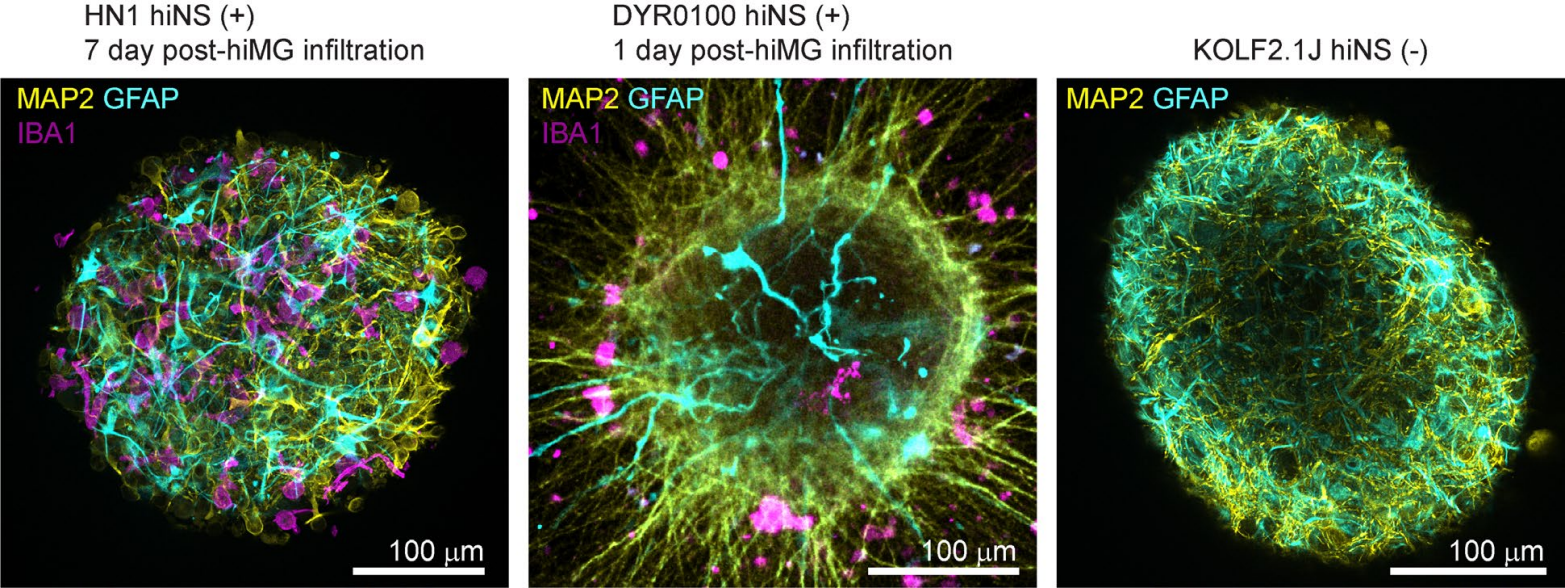
Immunofluorescence staining examples of human induced pluripotent stem cell (iPSC)-derived 3D neurosphere (hiNS) generated from different iPSC lines and two independent laboratories. Proof of concept immunofluorescence microscopy images for MAP2 (yellow), GFAP (cyan), and IBA1 (magenta) of HN1 hiNS (+) (including hiMG, 7 days post-infiltration) and DYR0100 hiNS (+) (including hiMG, 1 day post-infiltration). An additional immunostaining for MAP2 and GFAP in KOLF2.1J hiNS(-) (in the absence of hiMG) shows neuronal and astrocytic morphology in the absence of microglia.

**Critical:** Supplement NSM with IL-34/TGF-b (same concentration as used in MDM) after adding microglia to neurosphere cultures.

g. **To the plated neurospheres**, with a multichannel pipette speed set to slow, remove 120 mL of media from each well.

h. Prepare microglia in the required amount of 150 mL of NSM/well + 20 ng/mL of BDNF and GDNF + IL-34/TGF-b to reach a final count of 3 × 10^4^ microglia/neurosphere, e.g., for a full 48-well plate, add 1.44 × 10^6^ (3 × 10^4^ microglia × 48) to 7.2 mL (150 mL × 48) of NSM + 20 ng/mL of BDNF and GDNF + IL-34/TGF-b.

i. Incubate at 37 °C with 5% CO_2_. Repeat ~37% media changes with the above media every 48–72 h until DIV 60.

j. At DIV 60, neurospheres can be fixed for subsequent immunofluorescence staining. To label the major cell types present in hiNS, we recommend staining for MAP2 (for neurons), GFAP (for astrocytes), and IBA1 (for microglia) (Figure 3). Detailed immunofluorescence staining protocols are provided in the original publication and are not provided here, as the differentiation protocol is the focus of this article [14].

## Data analysis

Details about data analysis using our hiNS protocol can be found in our original article (see Statistical Analysis, Methods) [14].

We recommend at least 6–8 individual hiNS per group for adequate statistical comparisons.

## Validation of protocol

This protocol (or parts of it) has been used and validated in the following research article:

- Wendt et al. [14]. A 3D human iPSC-derived multi-cell type neurosphere system to model cellular responses to chronic amyloidosis. *J Neuroinflammation*.

## General notes and troubleshooting

**General notes**

1. First media change following first attachment of NPCs and iPSCs was found to optimally be performed at 12 h post-plating; however, this can be extended for up to 24 h if 12 h is not feasible.

**Troubleshooting**

Problem 1: EB formation is inconsistent, and too many iPSCs accumulate in the AggreWell plates.

Possible causes: Too many cells used, or the iPSC line is proliferating too much.

Solution: Reduce the number of iPSCs for EB seeding.

Problem 2: Microglia (hiMG) do not readily infiltrate into hiNS.

Possible cause: Early harvest of macrophage precursors from T75 flasks results in insufficient differentiation of hiMG.

Solution: Only harvest macrophage precursors after day 42 to ensure their maturity.

Problem 3: hiNS accumulate and aggregate at the edge of the Petri dish (DIV 7–40), reducing the total hiNS numbers available for experiments.

Possible cause: Petri dish dimensions or orbital shaker settings are not ideal.

Solution: Strictly use dimensions of Petri dishes presented in this protocol (9 cm culture dish) and use Celltron laboratory shaker at 65 rpm (or equivalent when using a different orbital shaker).
